# Draft genomes of two *Lethrus* species

**DOI:** 10.1038/s41597-026-06978-x

**Published:** 2026-03-05

**Authors:** Nikoletta Andrea Nagy, Levente Laczkó, Csongor Freytag, Renáta Bőkényné Tóth, Szabolcs Vencel Nagy, Gábor Sramkó, Zoltán Barta

**Affiliations:** 1 Department of Planetary Health, One Health Institute, Faculty of Health Sciences, University of Debrecen, Debrecen, Hungary; 2 HUN-REN–UD Behavioural Ecology Research Group, Department of Evolutionary Zoology, University of Debrecen, Debrecen, Hungary; 3 Department of Bioinformatics, One Health Institute, Faculty of Health Sciences, University of Debrecen, Debrecen, Hungary; 4 HUN-REN–UD Conservation Biology Research Group, University of Debrecen, Debrecen, Hungary; 5 Department of Infection Control and Hospital Epidemiology, One Health Institute, Faculty of Health Sciences, University of Debrecen, Debrecen, Hungary; 6 Pál Juhász-Nagy Doctoral School of Biology and Environmental Sciences, University of Debrecen, Debrecen, Hungary; 7 Centre for Metagenomics, University of Debrecen, Debrecen, Hungary; 8 Department of Botany, University of Debrecen, Debrecen, Hungary; 9 Department of Evolutionary Zoology and Human Biology, University of Debrecen, Debrecen, Hungary

**Keywords:** Genome, Evolutionary genetics

## Abstract

The superfamily Scarabaeoidae is a species-rich and diverse group within the order Coleoptera. The members of this taxon are of interest due to the diversity of their feeding and mating behaviour, and their ecological importance. Despite the size of the superfamily, only a few genomes have been published, leaving a large gap in our understanding of the evolution of these beetles. To reduce this gap, we generated third-generation sequencing data to describe the first genome assembly of *Lethrus scoparius* and to improve the assembly of *Lethrus apterus*. The genome of *L. scoparius* consists of 2,873 contigs with an N50 value of 301,243 bp. BUSCO analysis revealed 98.1% complete ortholog hits in the Endopterygota ortholog database. For the *L. apterus* genome, we were able to assemble 886 scaffolds with an N50 value of 1,378,308 bp and a complete BUSCO hit of 96.8%. We assigned functions to 15,252 genes in *L. scoparius* and 15,520 in *L. apterus*. These genomes may contribute to understanding the evolution of the superfamily.

## Background & Summary

The superfamily Scarabaeoidea - consisting of almost 42,000 species - is one of the most species-rich taxa within the order Coleoptera^1^. Species of the superfamily show great diversity in their morphology, behaviour and diet, which is due to coevolution with angiosperms and mammals as well as various types of selection pressures^2^. In addition, parental care is widespread among members of the superfamily, especially in the families Geotrupidae, Passalidae and Scarabaeidae^3^, and is often associated with nest building, with both behaviours occurring in different forms depending on which parent contributes, the division of labour and the structure of the nests^4^. In recent years, geotrupids and scarabids have gained increasing attention due to their grazing-connected lifestyle and ecological importance^5–8^. Despite an elevated research interest in the behaviour and life history strategies of these species, not much attention has been paid to their genetic background. To date, genomes are publicly available for only 42 of more than 35,000 scarabid, six out of 1,848 lucanid and two of 500 geotrupid species^1^ in the National Center for Biotechnology Information (NCBI) database (accessed 13 August 2025), leaving a gap in knowledge about the evolution of the superfamily, especially for the smaller families such as the Geotrupidae.

The largest genus within the family Geotrupidae is *Lethrus* with more than 130 species^9^. All species are flightless and prefer open habitats in the Palaearctic region^10^. The genus is unique within the family as its species feed on plant leaves and shoots rather than dung^11^. The genus *Lethrus* exhibits its highest species diversity in Middle Asia, where both allopatric and sympatric species occur^10^. One of these species is *Lethrus scoparius* Fischer von Waldheim, 1820, which has sympatric populations with other *Lethrus* species in the Western Tien Shan in Kazakhstan^10^. The western range of the genus is mainly characterised by allopatric species^10^, among which *Lethrus apterus* Laxmann, 1770^12^ has the largest distribution, a species that has been studied extensively, providing important results on its life history and physiology^13^, its genetic diversity^12^, and parental behaviour^14,15^ as well as its genetic background^16,17^. In addition, the draft genome of the species has been published^16^, although the assembly is highly fragmented, making it less reliable for genomic studies, e.g. reference-based transcriptome analyses. The variance in the distribution ranges of these species and the limited mobility of the beetles due to their inability to fly make *Lethrus* species great sources for studies on the genetic background of the speciation processes.

Here we present the first genome assembly of *Lethrus scoparius* and an improved version of the genome of *Lethrus apterus*. Both assemblies were constructed using Oxford Nanopore long read sequencing data, which greatly improves the quality and contiguity of *de novo* genomes, as can be seen in the case of the second version of the *L. apterus* genome. The draft genome of *L. scoparius* has a size of 266.04 Mbp and consists of 2,873 scaffolds with an N50 value of 301 kbp. The genome has a high gene completeness, i.e. 98.1% complete BUSCOs (96.6% single-copy and 1.5% duplicated). Using *ab initio* and homology-based methods, we predicted a total of 23,109 genes, of which 15,252 had a functional annotation. In the case of *L. apterus*, we combined the previously available short read dataset^16^ with the long read data and transcriptome reads to improve the contiguity and completeness of the genome. Our improved assembly resulted in a 293.02 Mbp long genome with 886 scaffolds and an N50 value of 1.38 Mbp, with which we constructed a much more contiguous genome than previously available. BUSCO analysis resulted in 96.8% complete genes (94.1% single-copy and 2.7% duplicated). For annotation, we combined *ab initio*, homology-based and evidence-based approaches and predicted a total of 16,631 genes. Functional annotation resulted in a final gene set of 15,520 genes. Comparing the two genome sequences, we found that 96.02% of the *L. scoparius* genome could be matched to the improved genome of *L. apterus* and we found 9,149 common orthologue clusters between the annotated genes of the two species. These genomes may contribute to future research on speciation processes within the genus and the evolution of parental care, nest building and feeding behaviour within the superfamily Scarabaeoidea, particularly the occurrence of phytophagy in a predominantly dung-feeding family.

## Methods

### Sample collection

We collected one male specimen of *Lethrus scoparius* on May 01, 2023, in Tian Shan, Kazakhstan (coordinates: 42°23.96′N 70°27.66′E) and which we stored in 96% ethanol on 4 °C until DNA isolation. For *L. apterus*, we collected one female individual on 09 May 2022, near the village of Susa, Hungary (coordinates: 48°16′27″N, 20°15′08″E). Northern Hungarian Inspectorate for Environment Protection and Nature Conservation approved the sample collection, *L. apterus* being a protected species in Hungary (No. 9007-8/2014).

### DNA isolation and sequencing

We used the whole body of both beetles for extracting high molecular weight DNA. We followed a conventional DNA isolation method, which consisted of a cell lysis step at 55 °C for two hours, where the lysis buffer contained a final concentration of 3 mM CaCl2, 2% sodium dodecyl sulfate (SDS), 40 mM dithiothreitol (DTT), 250 μg/ml proteinase K, 100 mM Tris buffer (pH = 8.0) and 100 mM NaCl^18^. We then centrifuged the samples at 14,000 *g* for 1 minute and transferred the supernatant to a clean tube to minimize chitin contamination during the following steps. We added 15 μl (10 mg/ml) RNase A (Roche, Switzerland) and further incubated the samples at 37 °C for 10 minutes. We continued by adding 0.5 volume of 7.5 M ammonium acetate and incubating at 4 °C for 10 minutes, then added 0.5 volume of a chloroform-isoamyl alcohol mixture (24:1) and incubated at room temperature for 10 minutes. This was followed by a centrifugation step at 10,000 *g* for 3 minutes after which we carefully transferred the supernatant to a clean tube. We added 1 volume of room temperature isopropanol and incubated the samples at 4 °C for 15 minutes. For pelletisation, we centrifuged the samples at 10,000 *g* for 3 minutes and then carefully removed the supernatant. We washed the pellets with 1 volume of room temperature 70% ethanol and centrifuged at 10,000 g for 3 minutes in between. Finally, we air dried the pellets and dissolved them in 65 μl of 10 mM Tris-HCl (pH = 8.0).

We measured DNA concentration and purity on a NABI UV/Vis Nano Spectrophotometer (MicroDigital Co., Ltd., Korea) and assessed DNA integrity by TBE agarose gel (1%) electrophoresis.

Before sequencing, we treated the isolates with RNase by adding 1 μl of RNase Cocktail™ Enzyme Mix (0.1 U RNase A and 4 U Rnase T1) (Invitrogen, USA) and incubating it at 37 °C for 30 minutes. We purified the samples with 0.6 volumes of Ampure XP (Beckman Coulter, USA) and removed short (<3–5 kbp) DNA fragments with 0.64 volumes of Long Fragment Buffer (Oxford Nanopore Technologies, UK) following the manufacturers’ protocols. We dissolved the DNA in 20 μl of nuclease-free water (Omega Bio-tek, Inc., USA). As a final step before library preparation, we checked the concentration and purity of the samples with a NanoDrop One (Thermo Fisher Scientific Inc., USA), a Qubit 4 Fluorometer (Thermo Fisher Scientific Inc., USA) with the 1x dsDNA High Sensitivity Kit (Thermo Fisher Scientific Inc., USA) and TBE agarose gel electrophoresis (1%).

We constructed the long read sequencing libraries with 1 μg of genomic DNA using the Ligation Sequencing Kit V14 (SQK-LSK114) (Oxford Nanopore Technologies, UK) following on the manufacturer’s instructions. We sequenced the final libraries on a MinION Mk1C device using an R10.4 flow cell (FLO-MIN114). Sequencing of *L. scoparius* resulted in a total of 7.28 Gbp long read data with an N50 value of 3,580 bp, whereas the sequencing of *L. apterus* yielded 9.16 Gbp of long read data with an N50 of 4,193 bp.

### Genome assembly

In both species, we first checked the quality of the raw reads with the MinIONQC R script^19^. We removed the DNA control strand with NanoLyse 1.2.0^20^ and the low-quality reads from the long read sequencing data using NanoFilt 2.8.0^20^. For this, we set the minimum mean quality score to eight, trimmed 50 bp from both ends of the reads to remove adapter contamination and discarded all reads shorter than 500 bp. For the assembly of the *L. apterus* genome, we completed the long read dataset with the raw Illumina short reads of the two females used to create the first draft genome of the species^17^ that were collected from the same population as our current sample (Table 1). After checking the quality of the Illumina reads with FastQC, we removed the adapter sequences and low-quality bases using fastp 0.20.1^21^. We set the 5′ and 3′ sliding window mean quality scores to 15 with a window size of 10, the minimum read length to 50 bp, we enabled the polyX trimming and the auto-detection of paired-end adapter sequences. Then we corrected for the sequence errors originating from the individual variance of the two specimens using Bloocoo 1.0.6^22^ with default settings. The filtering steps resulted in 2,751,077 long reads corresponding to 6.79 Gbp of data for *L. scoparius* (read N50: 4,124 bp). For *L. apterus*, we retained 2,589,368 long reads with a base count of 7.1 Gbp and N50 of 4,353 bp, and 34,707,186 short reads with a base count of 7.83 Gbp.

**Table 1:** Raw sequencing data used for the assembly and annotation of the genomes of *Lethrus scoparius* and *Lethrus apterus*.

Species	SRA accession number	Sequencing platform	Sequence type	Usage
*L. scoparius*	SRR28464392^60^	Oxford Nanopore	whole genome long read	genome assembly
*L. apterus*	SRR34367697^62^	Oxford Nanopore	whole genome long read	genome assembly
	SRR13594314^64^	Illumina HiSeq. 2500	whole genome PE125 short read	
	SRR13594315^65^			
	SRR30892904^66^	Illumina HiSeq. 4000	RNA-seq PE150 short read	genome annotation
	SRR30892909^67^			
	SRR30903940^68^			
	SRR30903941^69^			
	SRR30903979^70^			
	SRR30903980^71^			
	SRR30904354^72^			
	SRR30904355^73^			
	SRR30909762^74^			
	SRR30909765^75^			

**Fig. 1:**
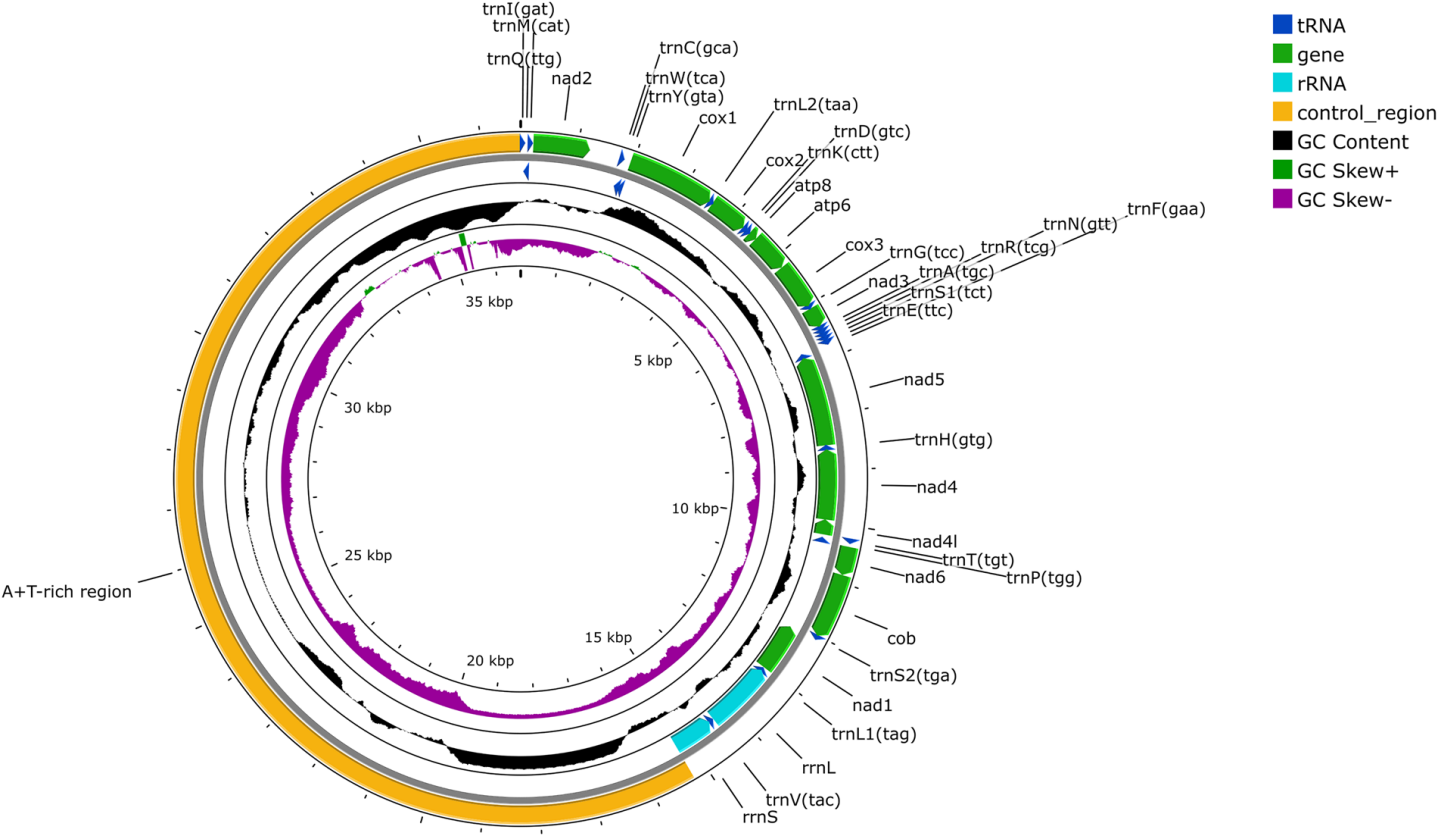
Mitochondrial genome of *Lethrus apterus* visualized by Proksee.

We first assembled the mitochondrial genomes of both species. The description of the assembly and annotation of the mitogenome of *L. scoparius* has been published in the superfamily-level phylogenetic study of Buban *et al*.^23^. To construct the complete mitochondrial genome of *L. apterus*, we aligned the long reads to the publicly available incomplete mitochondrion of *L. apterus* (GenBank accession number: BK067253) using minimap 2.17^24^. We then selected the reads that covered more than 90% of the reference sequence and assembled one circular contig using Flye 2.9^25^. We increased the accuracy of the sequence with racon 4.1.10^26^ and medaka 1.8.1^27^ with the model r1041_e82_400bps_sup_g615 using the long reads, resulting in a 36,388 bp long mitogenome. We ran MITOS 2.1.9^28^ to annotate the genes of the mitochondrial genome and then used Proksee^29^ for visualisation (Fig. 1). The arrangement of genes in the mitogenome of *L. apterus* did not differ from that of *L. scoparius*^23^.

To avoid contamination of the nuclear genome by sequences of mitochondrial origin, we aligned the long reads using minimap 2.17^24^ and the short reads with BWA 0.7.17^30^ to the assembled mitogenomes. Of the long read mappings, we discarded reads that covered more than 95% of the reference sequence. This threshold provided us the long reads not originating from mitochondrial genomes but did not exclude the nuclear mitochondrial DNA segments (NUMT) which may be present in the nuclear genomes. For the short reads, we removed all aligned reads using samtools 1.10^31^. This step yielded 6.76 Gbp (N50: 4,135 bp) of long read data for *L. scoparius* and 7.08 Gbp of long read (N50: 4,363 bp) and 7.7 Gbp of paired-end short-read data in the case of *L. apterus*. For genome size estimation, we calculated the 21-mer frequency distribution of the long read datasets with KMC 3.1.1^32^ and used the frequency histogram as input for GenomeScope2^33^. In the case of *L. scoparius*, the estimated genome size was 234.51 Mbp with a heterozygosity rate of 0.94% and a unique k-mer frequency of 80.8%. For *L. apterus*, GenomeScope2 predicted a genome size of 261.54 Mbp, the heterozygosity rate to be 0.09% and 77.1% of unique k-mer frequency.

Due to the different sequencing data, we assembled the two nuclear genomes using slightly different approaches, as detailed below. For *L. scoparius*, we only had long read data available, with which we assembled the initial genome using Flye 2.9^25^ with an estimated genome size of 300 Mbp and 30-fold assembly coverage. We set the estimated genome size as an intermediate value between the prediction of GenomeScope2 and the size of the publicly available geotrupid species (*Geotrupes spiniger*^34^ – 580.6 Mbp and *Lethrus apterus*^35^ – 286.9 Mbp). We polished the sequence of the initial genome with racon 4.1.10^26^ and medaka 1.8.1^27^ using the same method as for the mitochondrial genome. To remove false duplicates originating from the unresolved heterozygosity in the genome, we first ran the create_pseudohaploid.sh command from pseudohaploid^36^ which reduced the number of contigs from 5,529 to 5,088. To check whether there still are duplicated sequences, we applied redundans 0.11^37^ without scaffolding and gap closing. We tested different identity (0.60, 0.65, 0.70, 0.75, 0.80, 0.85, 0.90, 0.95, 1.0) and overlap (0.80, 0.85, 0.90, 0.95, 1.0) parameters to find the optimal values. The best combination of these two values was 0.6 identity and 0.8 overlap, based on the number of contigs (3,303) and the complete BUSCO score (98.4%). We then performed another polishing step using the same method as described above. Finally, we removed taxonomic contaminants by running BERTax 0.1^38^ to exclude sequences not derived from Arthropoda. The final decontaminated genome assembly for *L. scoparius* was 266.04 Mbp long and consisted of 2,873 contigs with an N50 value of 301,243 bp. The results of the BUSCO 5.2.2^39^ analysis using the endopterygota_odb10 database showed that the genome was very complete with a complete BUSCO score of 98.1% of which 1.5% were duplicated, and 1.2% were missing (Table 2).

**Table 2:** Assessment of contiguity and completeness of the final assemblies and annotations of the genomes of *Lethrus scoparius* and *Lethrus apterus* as estimated by QUAST and BUSCO in genome and proteome mode using the endopterygota_odb10 database.

	*L. scoparius* final genome	*L. apterus* improved genome	*L. apterus* GCA_018397195.1
# contigs (> = 0 bp)	2,873	886	66,933
# contigs (> = 1000 bp)	2,818	852	44,921
# contigs (> = 5000 bp)	2,150	577	17,982
# contigs (> = 10000 bp)	1,845	504	7,537
# contigs (> = 25000 bp)	1,457	431	949
# contigs (> = 50000 bp)	1,052	390	70
Total length (> = 0 bp)	266,042,793	293,017,777	286,931,630
Total length (> = 1000 bp)	266,003,785	292,996,002	271,771,675
Total length (> = 5000 bp)	264,087,979	292,208,261	203,473,167
Total length (> = 10000 bp)	261,816,446	291,701,319	129,803,375
Total length (> = 25000 bp)	255,343,032	290,514,697	32,716,838
Total length (> = 50000 bp)	240,812,982	288,975,250	4,448,004
# contigs	2,871	881	66,932
Largest contig	2,021,893	13,895,213	114,978
Total length	266,042,270	293,016,214	286,931,339
GC (%)	32.04	32.13	31.94
N50	301,243	1,378,308	8,902
N75	131,078	600,664	4,344
L50	231	48	8,985
L75	561	126	20,497
# N’s per 100 kbp	0.10	0.83	944.78
Complete BUSCOs (%)	2,084 (98.1)	2,056 (96.8)	1,985 (93.5)
Single-copy (%)	2,052 (96.6)	1,998 (94.1)	1,969 (92.7)
Duplicated (%)	32 (1.5)	58 (2.7)	16 (0.8)
Fragmented (%)	15 (0.7)	17 (0.8)	91 (4.3)
Missing (%)	25 (1.2)	51 (2.4)	48 (2.2)
Total number of BUSCOs	2124	2124	2124
Number of annotated genes – longest isoforms*	12,757	10,665	20,734
Complete BUSCOs (%)	2,000 (94.1)	1,980 (93.2)	1,915 (90.1)
Single-copy (%)	1,959 (92.2)	1,929 (90.8)	1,097 (51.6)
Duplicated (%)	41 (1.9)	51 (2.4)	818 (38.5)
Fragmented (%)	29 (1.4)	15 (0.7)	103 (4.8)
Missing (%)	95 (4.5)	129 (6.1)	106 (5.1)

*In the case of publicly available genome of *Lethrus apterus*, the number of annotated genes refers to the geneset published in Nagy *et al*.^17^.

In the case of *L. apterus*, we have created two initial assemblies. We used Flye 2.9^25^ to construct the genome from long read data only (estimated genome size 300 Mbp and 50-fold coverage), and we also performed a hybrid assembly from the combined long and short-read datasets using MaSuRCa 4.5.0^40^. After polishing the initial genomes, we used quickmerge 0.3^41^, with Flye as the first assembly and MaSuRCa as the second assembly. We polished the merged genome with racon 4.1.10^26^, medaka 1.8.1^27^, aligned the short reads to the genome and corrected for small mismatches with pilon 1.23^42^. We removed false duplicates with redundans 0.11^37^, as described for *L. scoparius*, and found 0.9 to be optimal threshold for both identity and overlap which decreased the number of contigs from 2,690 to 1,324. Prior to decontamination, we performed scaffolding based on transcriptome sequencing data from the species. For this purpose, we used all female samples from a previous study of ours on the seasonal changes in gene expression in this species^43^ (Table 1). We aligned the quality-filtered reads to the genome assembly using HISAT 2.1.0^44^ then joined the sequences with Rascaf 1.0.2^45^ to improve the contiguity of the genome. We then polished the sequence using the method described above and used BERTax 0.1^38^ for decontamination. The final assembly of *L. apterus* consisted of 886 scaffolds with a total size of 293.02 Mbp and an N50 value of 1,378,308 bp. Using the endopterygota_odb10 database, BUSCO 5.2.2^39^ analysis yielded 96.8% complete genes with 2.7% duplicate BUSCOs and 2.4% missing genes (Table 2).

### Genome annotation

The method for structural and functional annotation were largely identical for both *Lethrus* genomes. First, we performed the soft masking of the repetitive sequences with Red 2.0^46^, which resulted in 31.92% masked sequences for *L. scoparius* and 38.35% masked sequences for *L. apterus*. We then ran barrnap 0.9^47^ and ARAGORN 1.2.38^48^ to find the potential rRNA and tRNA genes in the assemblies, respectively. We found 28 rRNA and 362 tRNA genes in the genome of *L. scoparius* and 24 rRNA and 421 tRNA genes in *L. apterus*. Next, we used the BRAKER pipeline 3.0.2^49,50^ for gene prediction based on *ab initio* and homology-based methods for both beetles. In the case of *L. apterus*, we also used publicly available RNA-seq reads from adult female individuals (Table 1) for evidence-based gene prediction to improve the accuracy of the annotation. BRAKER used Augustus 3.5.0^51^ for *ab initio* gene prediction. We also used the Arthropoda_odb11 (https://bioinf.uni-greifswald.de/bioinf/partitioned_odb11/Arthropoda.fa.gz) to generate hints by ProtHint 2.6.0^52^. From these hints, GeneMark-EP 4.71_lic^52^ created a training dataset for Augustus for homology-based annotation. In the genome of *L. scoparius*, the *ab initio* method found 19,341 genes, while the homology-based prediction identified 25,449 genes. In *L. apterus*, the *ab initio* gene prediction yielded 14,704 genes, while the homology- and evidence-based method yielded 19,803 potentially protein-coding genes. We used the agat_sp_complement_annotations.pl script of Another Gtf/Gff Analysis Toolkit 1.4.1^53^ (AGAT) to merge the results of the different annotations by complementing the homology- and evidence-based results with the *ab initio* genes. We merged the predicted gene sets as the different structural annotation methods have distinct strengths. Homology-based prediction uses information from previously identified protein-coding genes and can identify conserved homologues based on sequences from other species, whereas the evidence-based method is useful for detecting different gene isoforms^54^. The *ab initio* method, by contrast, may be better suited for identifying novel proteins in a new assembly as it relies on general characteristics of the genes. These differences between prediction methods can result in varying numbers of genes, which may still include overlapping sequences. This step resulted in 26,399 genes (23,758 originating from homology-based and 2,641 from *ab initio* predictions) in *L. scoparius* and 22,042 genes (19,506 from the combined homology- and evidence-based and 2,536 from the *ab initio* gene sets) in *L. apterus*.

**Fig. 2:**
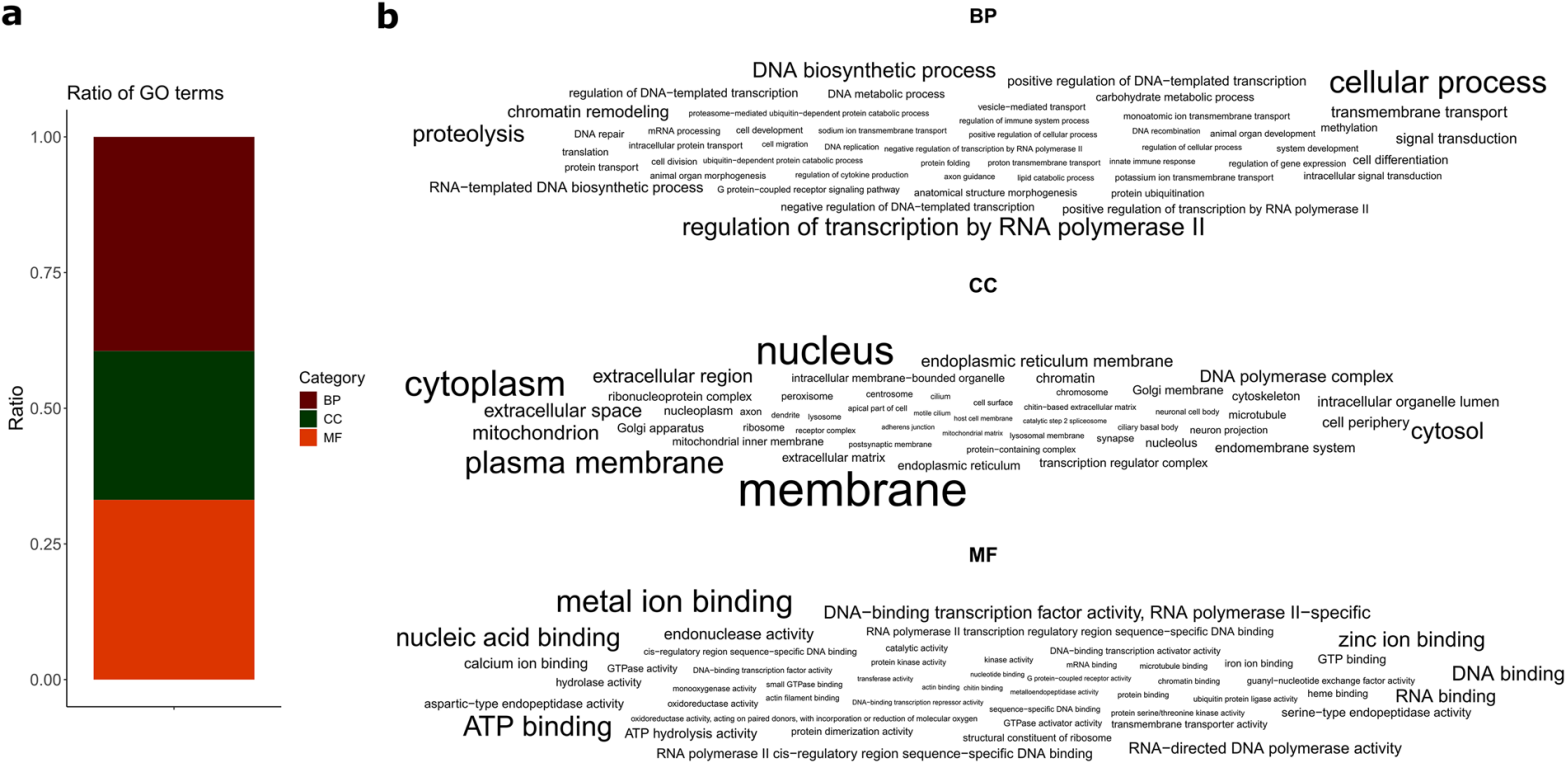
Results of the gene ontology (GO) annotation of the genome of *Lethrus scoparius*. (**a**) Ratio of the three main GO categories among the annotated genes (BP – biological process, CC – cellular component, MF – molecular function). (**b**) The 50 most frequent GO functions within the main categories represented as word clouds where the font size is proportional to the frequency.

**Fig. 3:**
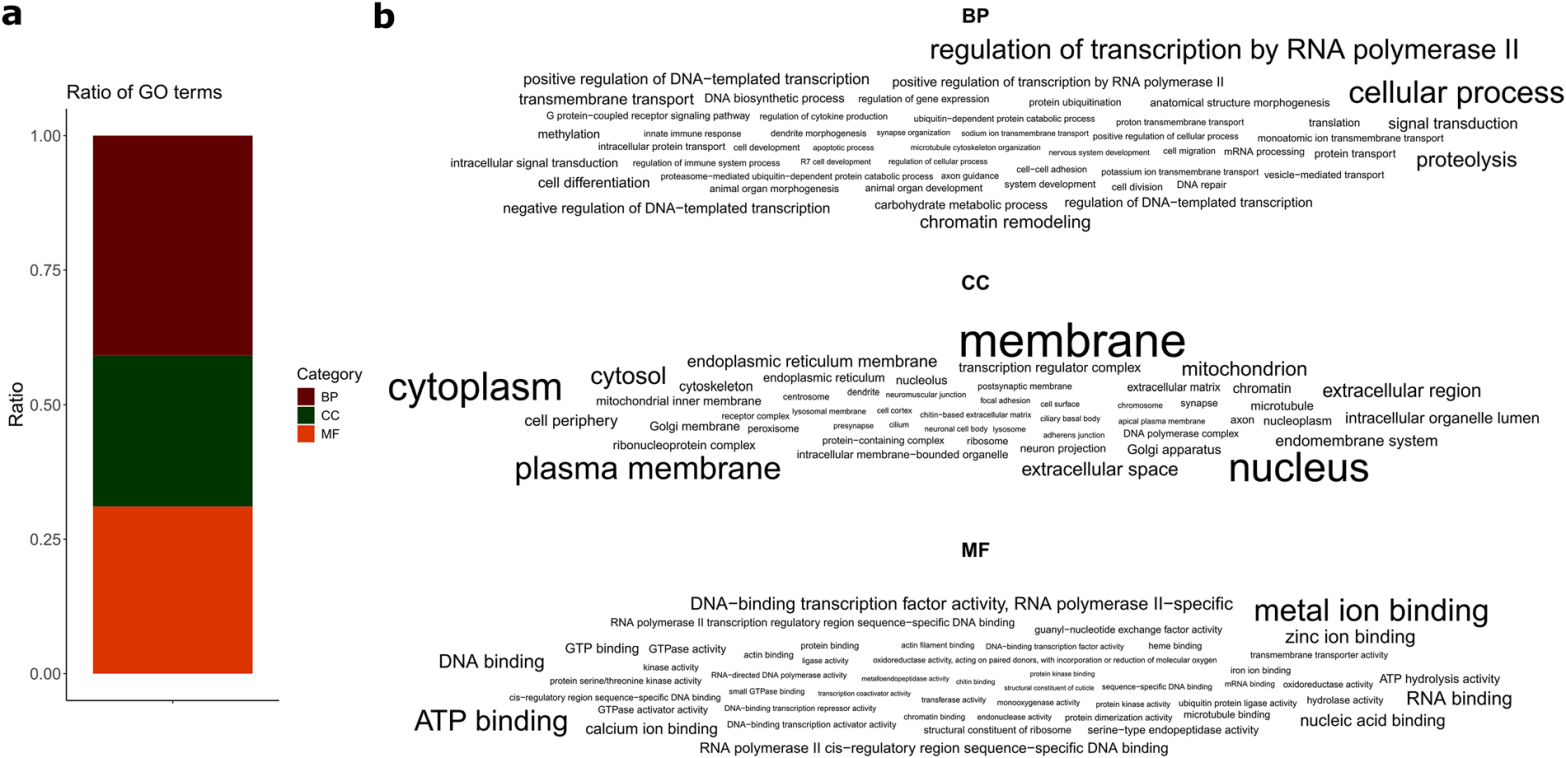
Results of the gene ontology (GO) annotation of the improved genome of *Lethrus apterus*. (**a**) Ratio of the three main GO categories among the annotated genes (BP – biological process, CC – cellular component, MF – molecular function). (**b**) The 50 most frequent GO functions within the main categories represented as word clouds where the font size is proportional to the frequency.

We used the agat_sp_extract_sequences.pl to obtain the corresponding protein sequences for all predicted protein-coding genes and submitted these sequences to the PANNZER web server^55^ (http://ekhidna2.biocenter.helsinki.fi/sanspanz/) for functional annotation. As a result, we found a total of 15,251 proteins in *L. scoparius* (57.78% of the predicted coding sequences) and 15,520 proteins in *L. apterus* (70.41% of the predicted coding sequences)^56^. Gene Ontology (GO) annotation showed that 39.46% of *L. scoparius* genes could be assigned to biological processes, with cellular processes, regulation of transcription by RNA polymerase II and proteolysis being the most frequent categories. 33.10% of the genes had a molecular function, with metal ion binding, ATP binding and nucleic acid binding being the most frequent annotations. The remaining genes (27.44%) belonged to the GO term cell components with the three most frequent functions being membrane, nucleus and cytoplasm (Fig. 2a,b). In the case of *L. apterus*, 40.86% of the genes were involved in biological processes, 31.08% had molecular functions and 28.07% were categorised as cell components (Fig. 3a). The most frequent GO terms within the main categories were identical to those identified in *L. scoparius* (Fig. 3b).

**Fig. 4:**
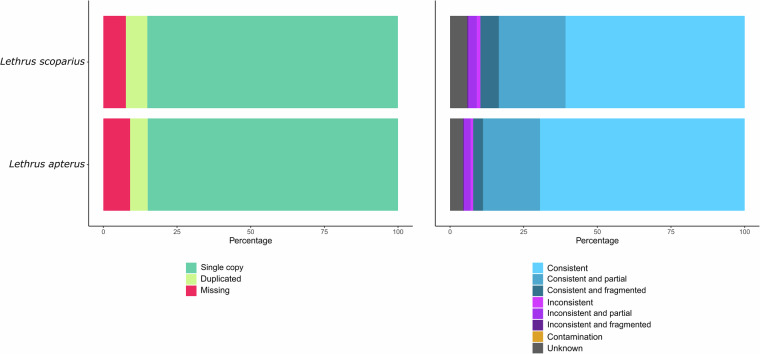
Gene content of the genome annotations of the two *Lethrus* species. (**a**) Proportion of single-copy, duplicated and missing genes of the Hierarchical Orthologous Groups (HOGs) characteristic to the Endopterygota clade. (**b**) Consistency assessment of the annotated genes.

Before checking the completeness of the gene sets, we filtered for the longest representative isoforms of the genes by applying agat_sp_keep_longest_isoform.pl to reduce the rate of false duplications. We then used BUSCO 5.2.2^39^ with the endopterygota_odb10 in proteome mode to examine the completeness of the functionally annotated gene set, and OMark 0.3.0^57^ (https://omark.omabrowser.org/home/ release 2024.06) with OMAmer 2.0.3 (database: All.Jul2023) to check for possible contamination and to visualise the consistency of the genes based on their homologs in other species. The 12,757 longest isoforms identified in the functionally annotated gene set of *L. scoparius* had a complete BUSCO score of 94.1% with 1.9% duplicate sequences and 4.5% missing genes (Table 2). According to OMark’s results, 92.35% of Hierarchical Orthologous Groups (HOGs) characteristic of the Endopterygota dataset were present in the proteome of *L. scoparius*, and consistency assessment revealed 89.71% consistent lineage placements but no contamination (Fig. 4). In the functionally annotated gene set of *L. apterus*, we found 10,665 longest isoforms with 93.2% complete BUSCOs - 2.4% duplicated - and 6.1% missing (Table 2). OMark results showed that 90.93% of the Endopterygota HOGs were present at the predicted protein level and the genes were consistently placed in the lineage at 92.16% without contamination (Fig. 4).

### Comparison of the genomes

Compared to the previous version^17^, we were able to improve the genome of *L. apterus* greatly. Our new method decreased the number of scaffolds from almost 67,000 to 886 and increased the N50 value from 8,902 bp to 1,378,308 bp. In addition, we improved the complete BUSCO score by 3.3% (from 93.5% to 96.8%, see Table 2). We used RagTag scaffold 2.1.0^58^ to check the sequence similarities of the *L. scoparius* genome and the previous genome version of *L. apterus* in comparison to the improved genome assembly of *L. apterus*. RagTag was able to map 255.44 Mbp (96.02%) of the *L. scoparius* genome to the *L. apterus* genome, showing that they are relatively closely related within the genus. However, only 257.92 Mbp (89.89%) of the publicly available genome of *L. apterus* could be aligned with the improved version, which could be the result of misassemblies due to the individual variations in the short-read dataset used for the previous version of assembly^17^ and the relatively high content of repetitive sequences in the genome.

**Fig. 5:**
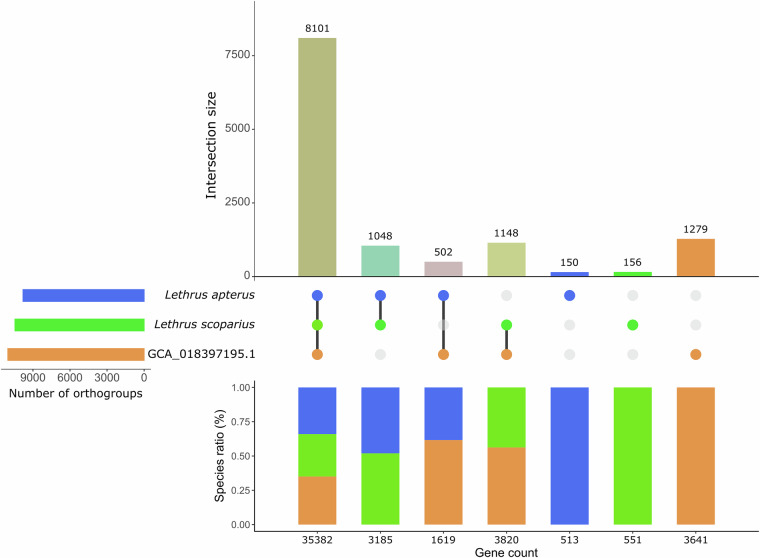
Comparison of the annotation of *Lethrus scoparius*, the improved and previous version (GCA_018397195.1^35^) of *Lethrus apterus* genome. Number of shared orthologous groups and species-specific groups in the three genomes are presented as upset plot (top). Number of genes belonging to the genomes in these orthogroups as percentages are presented in stacked bar charts (bottom) where the horizontal axis shows the size of the groups (total number of genes).

Although we found a lower number of annotated genes (15,520 genes compared to 20,734) in the new version of the genome, the proportion of complete BUSCOs in the proteome was higher in the new genome annotation (Table 2). We also submitted the final proteomes of the previous and new versions of the *L. apterus* genome together with the genome of *L. scoparius* to the OrthoVenn3 web server^59^ (https://orthovenn3.bioinfotoolkits.net/home) to visualise the similarities and differences between them. The results showed that most protein sequences could be assigned to ortholog groups present in all three genome annotations, and the publicly available version of the *L. apterus* genome had the highest number of assembly-specific genes and ortholog groups (Fig. 5). Comparing the genomes of the two species described here, we found that 1,304 orthogroups with 4,371 genes were species-specific in *L. scoparius*, while 651 orthogroups with 2,132 genes were specific to *L. apterus* (Fig. 5).

## Data Records

For the genome assembly of *L. scoparius*, all data described here belongs to the BioProject PRJNA1091353 in the NCBI Database. Raw data can be found under accession SRR28464392^60^ and the assembled genome under accession JBQXYL000000000^61^. We deposited the data of *L. apterus* described here under the BioProject PRJNA1285827 where the raw long read data is available under the accession number SRR34367697^62^ and the Whole Genome Shotgun project has been deposited at DDBJ/ENA/GenBank under the accession JBPULK000000000^63^. The annotated mitochondrial genome of *L. apterus* has been submitted as a separate record to the GenBank under the accession number BK071756. The version described in this paper is version JBPULK010000000^63^. The full annotation and the genome sequences of two species as well as the mitochondrial genome sequence of *L. apterus* are public in the Zenodo data repository under 10.5281/zenodo.16792786^56^.

## Technical Validation

We filtered the long reads using NanoLyse and NanoFilt and the genome and transcriptome short reads using fastp to remove DNA control strand, adapter sequences and low quality reads to achieve a low error rate and high contiguity and completeness of the assemblies. We discarded the mitochondrial reads before assembling the nuclear genome to avoid mitochondrial sequence contamination in the final assemblies. We used racon, medaka and pilon for sequence polishing to increase contiguity. We used redundans to reduce false duplicates in the genomes. We performed a decontamination step using BERTax to exclude sequences belonging to taxonomic groups that are not arthropods. After each step, we checked the contiguity and completeness of the assemblies with QUAST and BUSCO to compare the results of the applied changes to the genome sequences. For both species, we used *ab initio* and homology-based gene predictions and, in the case of *L. apterus*, we performed additional evidence-based predictions to obtain high-quality gene annotations. Using BUSCO and OMark, we checked the completeness of the genes, their consistency and possible contaminations in the final proteome of the species.

## Data Availability

Data belonging to *L. scoparius* are available in the NCBI database under BioProject PRJNA1091353. Raw data can be found under accession SRR28464392 and the assembled genome under accession JBQXYL000000000. Data of *L. apterus* belong to the BioProject PRJNA1285827 in the NCBI Database. Raw data can be found under accession SRR34367697 and the assembled genome under accession JBPULK000000000. The annotated mitochondrial genome of *L. apterus* is available under the GenBank accession number BK071756. The full annotation of the genomes, the genome sequences of the two species and the mitochondrial genome sequence of *L. apterus* are publicly available in the Zenodo data repository under 10.5281/zenodo.16792786.

## Code availability

We did not use any custom code in this study. We gave detailed description of the non-default parameters and versions of the tools used in this study in the Methods section.
